# 超高效液相色谱-三重四极杆质谱法测定大口黑鲈和草鱼胆汁中30种胆汁酸

**DOI:** 10.3724/SP.J.1123.2024.03004

**Published:** 2025-03-08

**Authors:** Lingwen MAO, Hao SUN, Haijie CHEN, Qianzhan YANG, Lan XU

**Affiliations:** 1.西南大学化学化工学院, 重庆 400715; 1. School of Chemistry and Chemical Engineering, Southwest University, Chongqing 400715, China; 2.西南大学水产学院, 重庆 400715; 2. College of Fisheries, Southwest University, Chongqing 400715, China; 3.岛津企业管理(中国)有限公司重庆分公司, 重庆 400010; 3. Shimadzu (China) Co., Ltd. Chongqing Branch, Chongqing 400010, China; 4.西南大学分析测试中心, 重庆 400715; 4. Analytical and Testing Center of Southwest University, Chongqing 400715, China

**Keywords:** 胆汁酸, 胆汁, 大口黑鲈, 草鱼, 超高效液相色谱-三重四极杆质谱, bile acids, bile, *Micropterus salmoides*, *Ctenopharyngodon idella*, ultra-high performance liquid chromatography-triple quadrupole mass spectrometry (UHPLC-MS/MS)

## Abstract

胆汁酸(bile acids, BAs)是胆汁的主要成分,在糖脂和胆固醇的代谢中具有重要的意义。大口黑鲈(*Micropterus salmoides*)和草鱼(*Ctenopharyngodon idella*)都是我国重要的淡水鱼养殖品种,明确两种鱼胆汁中胆汁酸谱可为该养殖领域提供数据支持。本研究利用超高效液相色谱-三重四极杆质谱开发了一种能同时检测30种胆汁酸的定性定量方法,并应用于大口黑鲈和草鱼胆汁中胆汁酸的种类和含量分析。方法优化了样品处理方法和仪器分析条件。胆汁样本经过离心后吸取上清液,以甲醇为提取溶剂涡旋混匀,经0.22 μm滤膜过滤后上机分析。采用Shim-pack Velox SP-C18色谱柱(100 mm×2.1 mm, 1.8 μm)进行分离,以0.01%冰乙酸水溶液-乙腈为流动相进行梯度洗脱,柱温50 ℃,进样量2 μL,采用电喷雾离子源(ESI)进行8种正离子、22种负离子的多反应监测(MRM)扫描。结果表明,30种胆汁酸在各自线性范围内线性良好,相关系数(*R*^2^)为0.9975~0.9997,回收率为72.3%~117.2%,相对标准偏差为0.46%~13.23%,方法的检出限(LOD)为0.01~0.75 ng/mL,定量限(LOQ)为0.02~2.28 ng/mL。利用所建立的方法对大口黑鲈和草鱼的胆汁进行检测,在大口黑鲈胆汁中成功检测出19种胆汁酸;在草鱼胆汁中成功检测出16种胆汁酸,大口黑鲈胆汁中有5种胆汁酸在草鱼胆汁中未检出,而草鱼胆汁中有2种胆汁酸在大口黑鲈胆汁中未检出。该方法简单快速,灵敏度和精确度高,适用于大口黑鲈和草鱼胆汁中30种胆汁酸的同时检测。

## Abstract (en)

Bile acids (BAs), the primary components of bile, play significant roles in sugar, lipid, and cholesterol metabolism. Normal BA metabolism maintains a dynamic equilibrium by regulating gut microbiota to effectively protect the liver and intestines, thereby sustaining overall health. Conversely, abnormal BA metabolism can cause intestinal tissue and liver damage, disruption of enterohepatic circulation homeostasis, dysbiosis of gut microbiota, and gastrointestinal and hepatic diseases. Although largemouth bass (*Micropterus salmoides*) and grass carp (*Ctenopharyngodon idella*) are important freshwater fish species in China, their BA profiles have not been fully characterized. Elucidating these profiles could provide valuable data to support the aquaculture industry.

In this study, a qualitative and quantitative method for the simultaneous determination of 30 BAs in the gallbladders of *M. salmoides* and *C. idella* was developed using ultra-high performance liquid chromatography-triple quadrupole mass spectrometry. The samples were centrifuged and then the supernatant was extracted, vortex-mixed with methanol, and filtered through a 0.22 μm membrane. Various mobile phase systems commonly used in liquid chromatography-mass spectrometry were investigated, including formic acid, acetic acid, ammonium formate, acetic acid-ammonium, and methanol-acetonitrile. Based on the response intensity, chromatographic separation, and peak shape of each substance, the optimal mobile phase was acetic acid and acetonitrile, and the concentration of acetic acid in water was optimized. To improve the ionization efficiency, the most effective ion scanning mode was selected by comparing the response intensities and peak conditions of each BA during mass spectrometry in positive and negative ion modes. Furthermore, the interface voltage for each BA and the ion source interface temperature were investigated to determine the optimal mass spectrometry conditions. Ultimately, separation was performed using a Shim-pack Velox SP-C18 column (100 mm×2.1 mm, 1.8 μm) with gradient elution of 0.01% acetic acid aqueous solution and acetonitrile, and the total analysis time was 14 min. The column temperature was 50 ℃ and the injection volume was 2 μL. Multiple reaction monitoring of 8 positive ions and 22 negative ions was carried out using electrospray ionization. Different series of mixed standard working solutions, prepared based on the response intensity of each BA, were used to construct standard curves. All 30 BAs exhibited good linearities within the investigated concentration ranges, with correlation coefficients (*R*^2^) of 0.9975-0.9997, indicating high accuracy. Spiked recoveries were 72.3%-117.2%, and the inter-day precisions were in the range of 0.46%-13.23%. The limits of detection and quantification were 0.01-0.75 ng/mL and 0.02-2.28 ng/mL, respectively, enabling the precise detection of low-concentration BAs. Using the established method, 19 BAs were successfully detected in the bile of *M. salmoides*, and 16 BAs were detected in the bile of *C. idella*, enriching the BA profiles of both species. Notably, five BAs present in the gallbladder of *M. salmoides* were undetected in the gallbladder of *C. idella*, whereas two BAs found in the gallbladder of *C. idella* were absent from the gallbladder of *M. salmoides*, demonstrating differences between the BA compositions of these species. The developed method is characterized by simplicity, speed, high sensitivity, and accuracy, harnessing the high-throughput advantages of ultra-high performance liquid chromatography-triple quadrupole mass spectrometry (UHPLC-MS/MS), and is suitable for the simultaneous detection of the 30 BAs in the gallbladders of *M. salmoides* and *C. idella*.

胆汁酸(bile acids, BAs)是一种类固醇化合物,主要存在于肠肝循环中,由肝脏中的胆固醇合成,并被肠道微生物群进一步代谢^[1]^。根据合成途径,胆汁酸可分为初级胆汁酸和次级胆汁酸。初级胆汁酸主要在肝细胞中合成,例如胆酸和鹅去氧胆酸,次级胆汁酸在肠道中合成,例如石胆酸和去氧胆酸^[2]^。胆汁酸代谢正常呈现动态平衡,可调节肠道菌群平衡,有效保护肝脏和肠道,维持身体健康^[3]^。而胆汁酸代谢异常则会导致肠道组织和肝脏损伤、肠肝循环稳态紊乱、肠道菌群失调,引起肠道和肝脏疾病^[4]^。

外源性胆汁酸可以维持葡萄糖稳态,改善鱼类的健康和生长性能,但这种影响会随着鱼类种类的不同而有所不同^[5]^。草鱼(*Ctenopharyngodon Idella*)是大宗淡水鱼中产量最多的品种,产量占到淡水养殖总产量的18.4%^[6]^。有研究表明石胆酸可以改变草鱼肝脏中的脂质代谢,并影响其肠道微粒菌群的组成^[7]^。而去氧胆酸能明显降低肝脏脂质含量和血清总胆固醇、甘油三酯、高密度和低密度脂蛋白胆固醇含量^[8]^。大口黑鲈(*Micropterus salmoides*)自引进后养殖规模得到迅速扩大,目前已成为我国主要的淡水鱼养殖品种之一,但大口黑鲈营养学研究起步较晚,各种营养参数还待完善,所需饲料需要进一步开发^[9]^。目前已有研究表明,在饲料中添加胆酸钠可以促进大口黑鲈鱼体肝糖原的合成、抑制肝脏和肌肉糖酵解^[10]^。而在饲料中添加去氧胆酸钠可以促进大口黑鲈的生长性能、胆汁酸合成的提高以及肌糖原的积累^[11]^。明确鱼体内胆汁酸种类以及含量有助于在养殖过程中减少各种疾病的发生,为水产养殖提供数据支持,而目前对于鱼胆汁中胆汁酸种类的研究较少,如采用超高效液相色谱-三重四极杆质谱法(UHPLC-MS/MS)同时检测草鱼胆汁酸中10种胆汁酸^[12]^,但该方法检测的胆汁酸种类较少。同时大口黑鲈胆汁酸谱的相关研究较少,因此建立多种胆汁酸的检测方法对于研究草鱼和大口黑鲈体内胆汁酸代谢有一定帮助。

胆汁酸的种类繁多、成分复杂,存在多种同分异构体^[13]^,增加了分析检测难度,目前应用于胆汁酸的检测方法有气相色谱法(GC)、高效液相色谱法(HPLC)、薄层色谱法(TLC)、分光光度法(SP)、气相色谱-质谱法(GC-MS)、液相色谱-质谱法(LC-MS)^[14,15]^。其中全扫描(SCAN)、选择离子监测(SIM)、选择反应监测(SRM)和多反应监测(MRM)模式有助于实现胆汁酸的精准定性和准确定量^[16]^。对比GC-MS,LC-MS具备无需衍生化的优点^[17]^,已被广泛用于人类和动物样本中胆汁酸的分离和定性定量检测^[18]^。

本研究通过UHPLC-MS/MS建立了大口黑鲈和草鱼胆汁中30种胆汁酸的检测方法。该方法操作简单、快速、灵敏度高,为两种鱼的胆汁酸相关研究提供了胆汁酸谱的数据参考。

## 1 实验部分

### 1.1 仪器、试剂与材料

LCMS-8060NX超高效液相色谱-三重四极杆质谱仪(日本Shimadzu公司); C18-60-SC实验室超纯水终端(重庆博创水处理设备有限公司); TUC-10H加热超声波清洗器、VM-T1涡旋混合器(上海泰坦科技股份有限公司); 1-14K高速冷冻离心机(德国Sigma公司); XPR 2/A百万分之一天平(瑞士Mettler Toledo公司)。

甲醇、乙腈(色谱纯,上海泰坦科技股份有限公司);甲酸(色谱纯,上海安谱实验科技股份有限公司);甲酸铵(色谱纯,上海迈瑞尔生化科技有限公司);乙酸铵(色谱纯,上海阿拉丁生化科技有限公司);冰乙酸(分析纯,上海易恩化学技术有限公司);无水乙醇(分析纯,重庆川东化工有限公司); 30种胆汁酸标准品见表1(上海安谱实验科技股份有限公司、上海源叶生物科技有限公司、上海泰坦科技股份有限公司)。实验所用大口黑鲈由西南大学水产学院培育,草鱼由北碚区农贸市场收集。两种鱼经消毒后无菌剥离胆囊组织,于-20 ℃避光保存。

**表1:** 30种胆汁酸的质谱参数

No.	Compound	Abbreviation	Retention time/min	ESI	Precursor ion (m/z)	Product ion (m/z)	Interface voltage/kV	Collision energy/V
1	tauro-α-muricholic acid (牛磺-α-鼠胆酸)	TαMCA	2.607	+	480.30	337.40	3	20
2	tauro-hyocholic acid (牛磺猪胆酸)	THCA	3.304	-	514.30	107.15	-2	-55
3	tauro-ursodeoxycholic acid (牛磺熊去氧胆酸)	TUDCA	3.623	-	498.60	498.60	-3	-31
4	tauro-hyodeoxycholic acid (牛磺猪去氧胆酸)	THDCA	3.761	-	498.20	124.20	-3	-32
5	tauro-cholic acid (牛磺胆酸)	TCA	3.811	+	480.30	462.40	3	18
6	tauro-chenodeoxycholic acid (牛磺鹅去氧胆酸)	TCDCA	4.863	-	498.60	498.60	-2	-32
7	tauro-deoxycholic acid (牛磺去氧胆酸)	TDCA	5.078	-	498.30	124.10	-2	-54
8	glyco-hyocholic acid (甘氨猪胆酸)	GHCA	5.531	-	464.25	74.20	-3	-41
9	7,12-keto-lithocholic acid (7,12-二酮石胆酸)	7,12KLCA	5.957	+	405.25	387.40	4	10
10	glyco-cholic acid (甘氨胆酸)	GCA	6.118	-	464.40	74.20	-2	-38
11	glyco-ursodeoxycholic acid (甘氨熊去氧胆酸)	GUDCA	6.135	-	448.25	74.15	-3	-35
12	dehydrocholic acid (去氢胆酸)	DHCA	6.162	+	403.30	385.45	3	11
13	glyco-hyodeoxycholic acid (甘氨猪去氧胆酸)	GHDCA	6.271	-	448.25	74.20	-2	-60
14	tauro-lithocholic acid (牛磺石胆酸)	TLCA	6.377	-	482.30	124.25	-2	-51
15	α-muricholic acid (α-鼠胆酸)	αMCA	6.619	+	373.35	355.50	4	14
16	β-muricholic acid (β-鼠胆酸)	βMCA	6.931	-	407.60	407.60	-3	-30
17	hyocholic acid (猪胆酸)	HCA	7.453	-	407.35	407.35	-3	-30
18	glyco-chenodeoxycholic acid (甘氨鹅去氧胆酸)	GCDCA	7.848	-	448.20	74.10	-2	-34
19	cholic acid (胆酸)	CA	7.931	-	407.25	289.55	-3	-40
20	glyco-deoxycholic acid (甘氨去氧胆酸)	GDCA	8.122	-	448.25	74.00	-3	-45
21	ursodeoxycholic acid (熊去氧胆酸)	UDCA	8.274	-	391.55	391.55	-3	-30
22	hyodeoxycholic acid (猪去氧胆酸)	HDCA	8.422	-	391.60	391.60	-3	-30
23	6-keto-lithocholic acid (6-酮石胆酸)	6KLCA	8.743	+	391.25	355.40	4	17
24	7-keto-lithocholic acid (7-酮石胆酸)	7KLCA	9.107	+	373.35	355.45	3	15
25	12-keto-lithocholic acid (12-酮石胆酸)	12KLCA	9.321	+	391.25	309.45	4	20
26	chenodeoxycholic acid (鹅去氧胆酸)	CDCA	10.124	-	391.55	391.55	-3	-30
27	deoxycholic acid (去氧胆酸)	DCA	10.353	-	391.20	345.55	-4	-35
28	glyco-lithocholic acid (甘氨石胆酸)	GLCA	10.533	-	432.35	74.20	-3	-33
29	obeticholic acid (奥贝胆酸)	OCA	11.621	-	419.30	401.60	-3	-33
30	lithocholic acid (石胆酸)	LCA	11.849	-	375.55	375.55	-3	-30

### 1.2 标准储备液配制

精密称取30种胆汁酸标准品适量,用甲醇超声溶解,配制成质量浓度为1 mg/mL的标准储备液,于-20 ℃冰箱中避光保存。根据实验需要用甲醇配制成不同浓度的混合标准工作液。

### 1.3 样品前处理

取胆囊组织适量,经水浴解冻后取1 mL混匀的胆汁样本于1.5 mL离心管中,以4000 r/min离心20 min,吸取100 μL上清液,加入900 μL甲醇涡旋振荡30 s,经0.22 μm滤膜过滤后上机分析。

### 1.4 分析条件

色谱柱:Shim-pack Velox SP-C18 (100 mm×2.1 mm, 1.8 μm);流动相A为0.01%冰乙酸水溶液,流动相B为乙腈。梯度洗脱程序:0~0.50 min, 20%B; 0.50~10.00 min, 20%B~55%B; 10.00~10.50 min, 55%B~100%B; 10.50~12.00 min, 100%B; 12.00~12.01 min, 100%B~20%B; 12.01~14.00 min, 20%B。流速为0.45 mL/min;柱温为50 ℃;进样量为2 μL。

采用电喷雾离子源(ESI),通过MRM模式进行正、负离子模式同时扫描;碰撞气为高纯氩气;雾化气为高纯氮气,流速2.0 L/min;干燥气为高纯氮气,流速5.0 L/min;加热气为空气,流速15 L/min;加热块温度:500 ℃;接口温度:350 ℃;脱溶剂温度:602 ℃;脱溶剂管(DL)温度:300 ℃。30种胆汁酸标准品的保留时间及质谱参数见表1。

数据分析通过LabSolutions Ver. 5.99软件进行处理。

## 2 结果与讨论

### 2.1 色谱条件的优化

次级胆汁酸大多为同分异构体,在低分辨质谱中难以得到质荷比的明显差异,因此在定性检测中对色谱分离条件要求高。为得到更好的分离效果,本研究考察了多种在液相色谱-质谱中常用的流动相体系,甲酸、冰乙酸、甲酸铵、乙酸铵水溶液搭配甲醇或乙腈。结果表明,有机相采用甲醇时,基线比采用乙腈时更高,响应更低,胆汁酸的整体出峰较晚,不符合快速检测的目的,因此选用乙腈作为有机相。考察5 mmol/L甲酸铵、5 mmol/L乙酸铵、0.1%甲酸水溶液和0.1%冰乙酸水溶液搭配乙腈作流动相对于胆汁酸色谱峰峰形和分离的影响。结果表明,当采用5 mmol/L甲酸铵水溶液-乙腈作为流动相时,胆汁酸响应强度较低,分离效果较差;采用5 mmol/L乙酸铵水溶液-乙腈作为流动相时,所有胆汁酸的响应强度均有所提高,分离效果相较于甲酸铵体系没有明显差异;采用0.1%甲酸水溶液-乙腈作为流动相时,分离效果较好,大部分胆汁酸响应强度有所提高,但TLCA的峰形变差,LCA的响应强度较低;采用0.1%冰乙酸水溶液-乙腈作为流动相时,分离效果较好,大部分胆汁酸响应强度对比甲酸体系无明显差距,而TLCA的峰形更尖锐,LCA的响应强度更高(见图1)。因此采用冰乙酸搭配乙腈作为流动相体系。

**图1:**
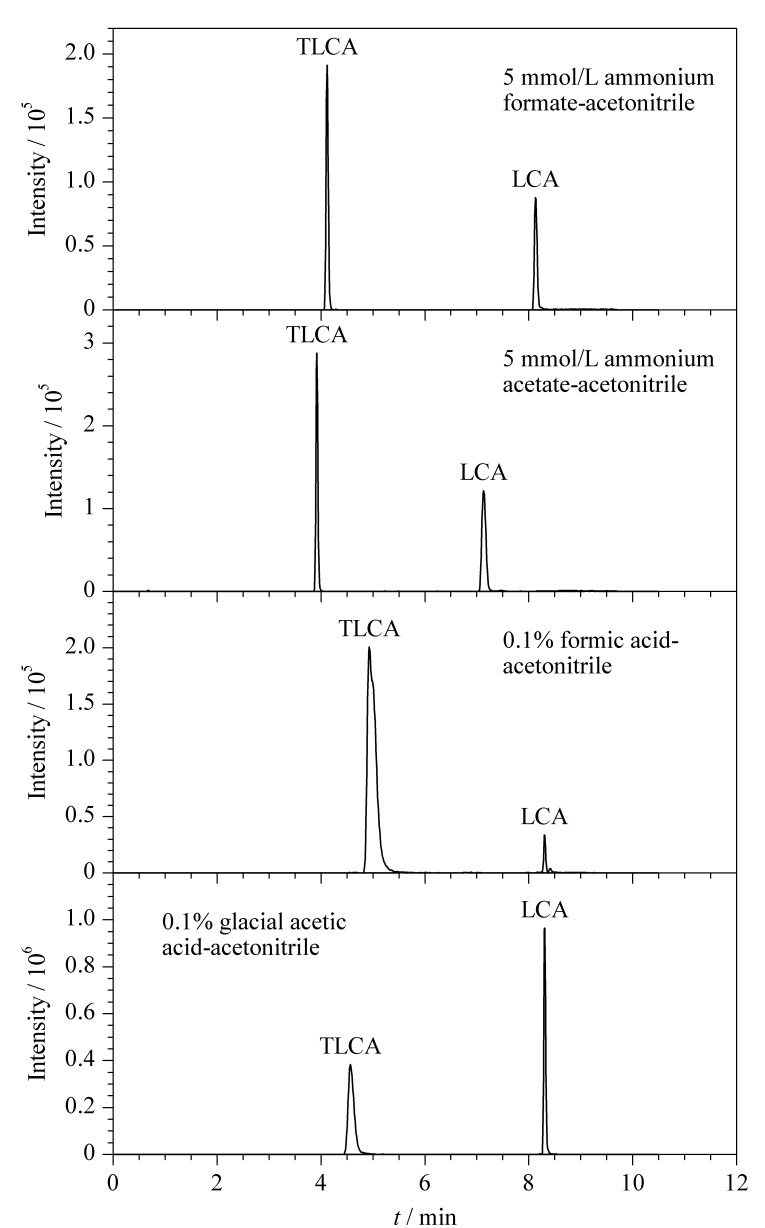
TLCA和LCA在4种流动相体系中的MRM色谱图

为了提升部分胆汁酸的响应强度,尝试改变水相中冰乙酸的体积分数(0.01%、0.05%、0.1%、0.15%),对比结果如图2所示。结果表明,随着添加冰乙酸体积分数的降低,胆汁酸的响应强度逐渐提高,0.01%冰乙酸水溶液的效果最佳,因此选择0.01%冰乙酸-乙腈作为最终的流动相体系。在优化的条件下,30种胆汁酸混合标准工作液的MRM色谱图见图3。

**图2:**
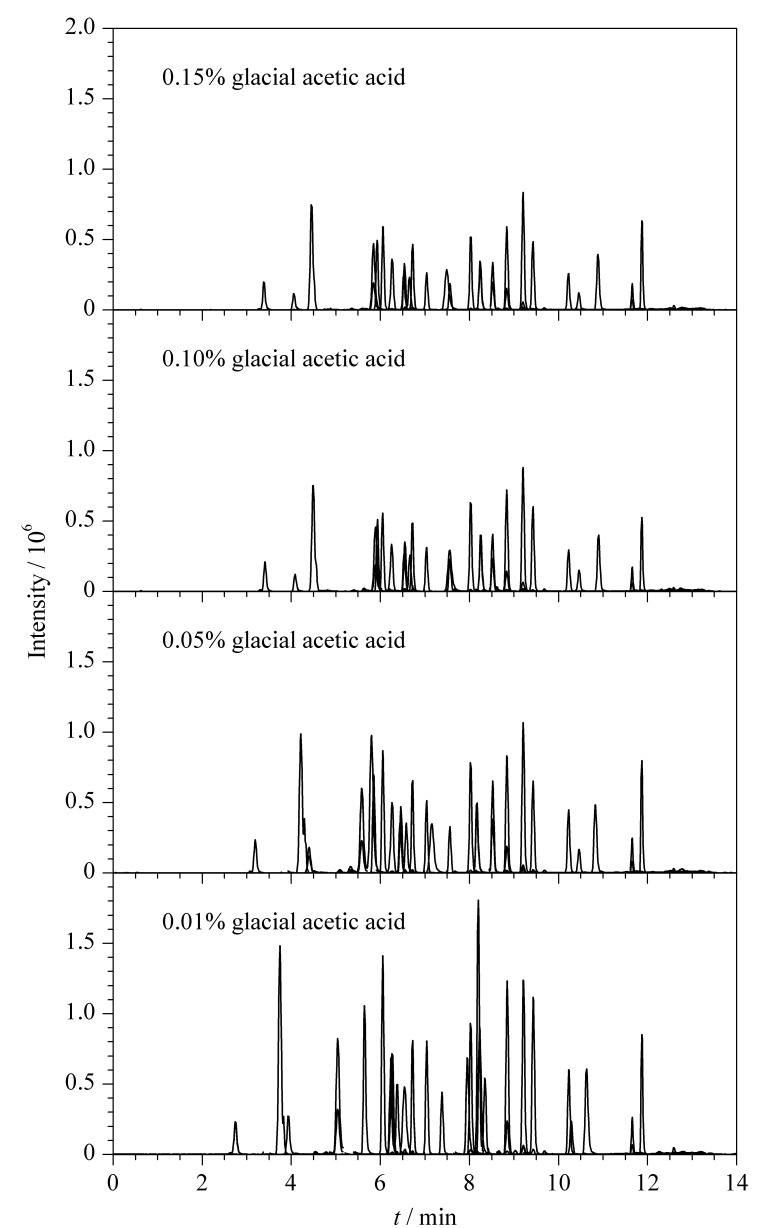
不同体积分数的冰乙酸对30种胆汁酸响应强度的影响

**图3:**
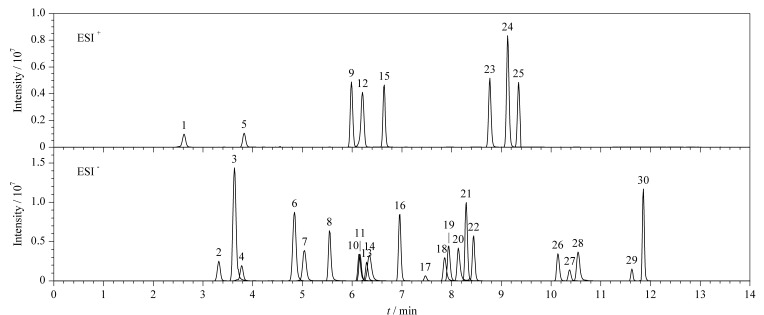
30种胆汁酸混合标准工作液在正、负离子模式下的MRM色谱图

### 2.2 质谱条件的优化

首先将30种胆汁酸的标准储备液用甲醇稀释至1 μg/mL和100 ng/mL两种浓度,将1 μg/mL的标准溶液分别在ESI^+^和ESI^-^两种模式下进行SCAN扫描,得到正、负离子模式下的前体离子,然后通过仪器自有的方法优化程序对100 ng/mL的混合标准溶液进行正、负离子模式下的质谱参数优化。连续进样6次,自动调整前体离子、产物离子、碰撞能量,得到每一种物质的基本离子对信息以及质谱参数,建立初始的MRM方法。再将30种胆汁酸的标准储备液稀释混合成100 ng/mL的混合标准工作液后进行MRM扫描,结果显示,6KLCA、7KLCA、12KLCA、7,12KLCA、GHCA、DHCA、TCA、TαMCA在ESI^+^下的响应强度比ESI^-^下的响应强度高,因此这8种胆汁酸在ESI^+^模式下检测,其他22种胆汁酸在ESI^-^模式下检测。确认离子扫描模式后单独调整每个MRM方法的接口电压(1~5 kV),通过响应强度的对比确定每一个MRM方法的最佳接口电压,结果见表1。

为提升胆汁酸的离子化效率,考察了200、250、300、350和400 ℃这5种不同接口温度下各胆汁酸的响应强度,其中*α*MCA、T*α*MCA、TCA、GCA、GHDCA、THCA、TUDCA的响应强度随接口温度改变而有明显变化,如图4所示。结果表明,随着接口温度的提高,7种胆汁酸的响应强度有明显增强,350 ℃时响应强度最高,而接口温度继续升至400 ℃后各胆汁酸的响应强度有所下降,因此选择350 ℃作为离子源的接口温度。

**图4:**
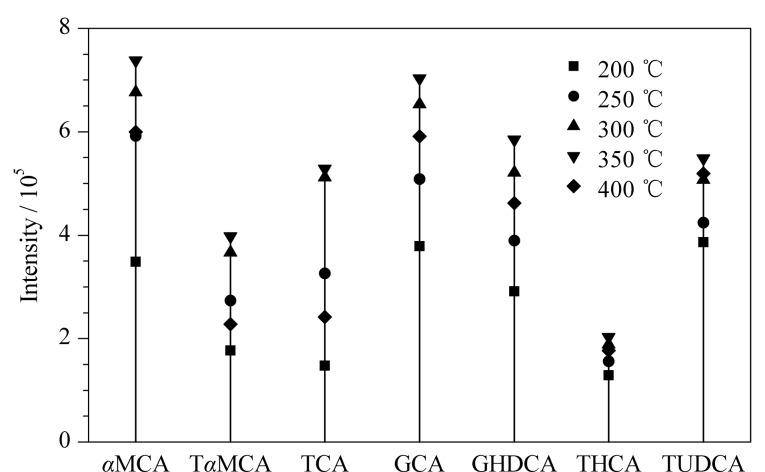
不同离子源接口温度下7种胆汁酸的响应强度

### 2.3 提取溶剂的选择

本研究考察了甲醇、乙腈、超纯水、无水乙醇4种常见提取溶剂对于胆汁酸定性、定量的影响。结果表明,4种提取溶剂对于大口黑鲈和草鱼胆汁的溶解性排序为甲醇>无水乙醇>超纯水>乙腈;其中乙腈作为提取溶剂时会有黑色胆汁样品析出,过滤膜后经乙腈再稀释依旧会有黑色胆汁样品析出,因此判断大口黑鲈和草鱼胆汁难溶于乙腈;而超纯水提取后的样品经4 ℃冰箱冷藏后,样品溶液会再次呈现浑浊状态,判断为胆汁内基质复杂,微生物有所衍生;甲醇以及无水乙醇作为提取溶剂具有较高的溶解度,并能保持溶解后样品溶液的稳定性。且甲醇作为提取溶剂时大部分胆汁酸的回收率优于无水乙醇。

### 2.4 线性范围、检出限与定量限

根据各胆汁酸的响应强度不同,精确配制30种不同质量浓度的胆汁酸混合标准工作液,稀释得到系列混合标准工作液上机进行MRM扫描。以各胆汁酸的质量浓度(*x*, ng/mL)为横坐标,以相对应的峰面积(*y*)为纵坐标,建立30种胆汁酸的标准曲线。结果显示,各胆汁酸在线性范围内的线性关系良好,相关系数(*R*^2^)为0.9975~0.9997(见表2),以3倍和10倍信噪比(*S/N*)所对应的质量浓度作为方法的检出限(LOD)和定量限(LOQ),方法的LOD为0.01~0.75 ng/mL, LOQ为0.02~2.28 ng/mL。

**表2:** 30种胆汁酸的回归方程、线性范围、相关系数、检出限、定量限、回收率和相对标准偏差

Compound	Regression equation	Linear range/ (ng/mL)		R^2^	LOD/ (ng/mL)	LOQ/ (ng/mL)	Recovery/%	RSD/%
TαMCA	y=2129.66x+10.902	2-	800	0.9994	0.75	2.28	102.2-103.9	2.44-9.50
THCA	y=10381.5x-130.457	1-	400	0.9983	0.28	0.85	90.5-95.9	0.96-4.86
TUDCA	y=547013x-4619.620	0.1-	60	0.9975	0.01	0.04	75.1-88.3	1.77-3.15
THDCA	y=12041.2x-179.399	0.5-	400	0.9979	0.12	0.37	73.6-83.4	1.19-1.65
TCA	y=2821.40x-134.858	1-	800	0.9991	0.26	0.79	74.5-94.0	3.43-13.23
TCDCA	y=131215x-6194.000	0.5-	200	0.9986	0.12	0.44	87.0-111.0	1.01-3.48
TDCA	y=17878.2x-913.608	0.5-	400	0.9994	0.34	1.02	102.7-111.0	0.77-5.20
GHCA	y=82864.7x-1743.410	0.25-	200	0.9985	0.03	0.05	76.9-93.9	0.78-1.81
7,12KLCA	y=14789.4x-1481.970	0.5-	400	0.9990	0.15	0.47	84.1-89.1	2.26-6.38
GCA	y=24040.8x-301.206	0.5-	400	0.9994	0.01	0.02	78.8-89.9	1.26-2.72
GUDCA	y=50287.9x+708.853	0.25-	200	0.9982	0.01	0.02	74.6-94.5	1.84-2.00
DHCA	y=15564.4x-829.542	0.5-	400	0.9987	0.27	0.83	87.5-101.1	1.22-8.94
GHDCA	y=18017.5x+265.523	0.5-	400	0.9981	0.01	0.04	84.7-92.0	1.65-1.95
TLCA	y=7362.54x-556.935	1-	800	0.9996	0.30	0.91	94.0-99.4	1.18-4.14
αMCA	y=26397.2x-719.050	0.5-	200	0.9976	0.23	0.70	81.5-83.1	0.76-4.24
βMCA	y=23837.7x+184.738	1-	800	0.9991	0.01	0.02	75.15-93.6	2.42-7.93
HCA	y=3071.45x+314.407	5-	2000	0.9983	0.35	1.07	72.3-96.0	3.46-12.70
GCDCA	y=25843.7x+428.628	0.5-	400	0.9992	0.01	0.03	75.5-86.1	0.84-2.41
CA	y=4233.44x-622.072	2-	1600	0.9997	0.03	0.13	101.0-112.0	1.49-5.60
GDCA	y=37281.8x-414.277	0.5-	400	0.9989	0.01	0.04	78.2-93.9	1.41-1.74
UDCA	y=37321.3x-4425.300	1-	800	0.9990	0.05	0.16	79.2-117.2	1.66-5.84
HDCA	y=16895.3x-1071.060	1-	800	0.9987	0.05	0.15	79.2-108.6	1.44-8.77
6KLCA	y=10713.2x+380.224	0.5-	400	0.9994	0.17	0.53	80.6-87.5	2.45-4.18
7KLCA	y=36288.5x-4209.580	0.5-	200	0.9993	0.28	0.85	75.5-83.3	2.06-3.11
12KLCA	y=11916.1x-753.243	0.5-	400	0.9983	0.44	1.33	83.3-91.5	2.70-4.27
CDCA	y=5365.63x-305.523	2.5-	2000	0.9996	0.17	0.53	75.0-99.5	1.10-5.99
DCA	y=416.149x-35.7764	10-	8000	0.9991	0.37	1.11	104.0-108.0	1.02-5.20
GLCA	y=29692.9x-2627.380	0.5-	400	0.9992	0.02	0.06	76.0-81.8	0.46-3.42
OCA	y=946.809x+291.909	8-	3200	0.9979	0.22	0.66	100.1-112.0	3.77-5.14
LCA	y=18248.4x+1515.640	1-	800	0.9976	0.08	0.25	75.1-113.0	6.37-10.19

*y*: peak area; *x*: mass concentration, ng/mL.

### 2.5 回收率与精密度

以质量浓度为400 ng/mL的胆汁酸为基准,将混合标准工作液用甲醇稀释成低(10 ng/mL)、中(25 ng/mL)、高(50 ng/mL)3个水平。取两种鱼的胆汁适量,用甲醇处理后加入混合标准工作液,每个水平重复检测6次,结果如表2所示,两种鱼胆汁的胆汁酸回收率为72.3%~117.2%,相对标准偏差为0.46%~13.23%。

### 2.6 胆汁样品检测

准备大口黑鲈和草鱼各18尾,并将每6尾同种鱼的胆汁混合成一组样本,准备3组平行试样,按1.3节步骤处理后上机进行MRM扫描,最终得出两种鱼胆汁中胆汁酸含量的数据如表3所示。

**表3:** 两种鱼胆汁中检出的胆汁酸含量(*n*=3)

Compound	Micropterus salmoides	Ctenopharyngodon idella
TαMCA	ND	ND
THCA	ND	19226.07±3603.93
TUDCA	ND	ND
THDCA	ND	ND
TCA	3991821.00±457739.50	56856.00±11186.00
TCDCA	5012600.00±1047645.00	111798.00±18736.00
TDCA	20168.41±5499.49	ND
GHCA	40.16±9.71	11.59±0.74
7,12KLCA	18.51±3.96	ND
GCA	2282.10±780.40	1229.00±242.00
GUDCA	ND	ND
DHCA	ND	ND
GHDCA	160.27±33.27	433.60±136.40
TLCA	75359.03±9053.84	14614.10±1434.10
αMCA	ND	ND
βMCA	ND	ND
HCA	ND	1406.00±247.93
GCDCA	1601.62±680.48	3630.05±191.81
CA	45939.10±12245.38	7944.93±1448.07
GDCA	101.45±9.42	204.85±14.41
UDCA	109.69±27.24	ND
HDCA	57.91±9.21	736.34±277.47
6KLCA	ND	ND
7KLCA	287.74±17.59	ND
12KLCA	2.78±0.82	ND
CDCA	81006.80±21096.39	11630.63±3435.87
DCA	5659.98±1829.69	1625.71±632.14
GLCA	7.53±0.27	31.72±13.38
OCA	ND	ND
LCA	7.03±2.58	3.96±1.41

ND: not detected.

结果显示,大口黑鲈胆汁中成功检出19种胆汁酸,草鱼胆汁中成功检出16种胆汁酸。从检测结果可知,两种鱼胆汁中CA、CDCA、TCDCA、TCA、TLCA含量较高。大口黑鲈胆汁中检出的12KLCA、7,12KLCA、UDCA、7KLCA和TDCA在草鱼胆汁中并未检出,而在草鱼胆汁中检出的THCA和HCA在大口黑鲈胆汁中并未检出。在大口黑鲈胆汁中成功检测出的胆汁酸种类比草鱼多,而且大口黑鲈胆汁中不同胆汁酸含量差异比草鱼大,这可能是两种鱼品种不同的原因,体内胆汁酸代谢和吸收有所区别。

## 3 结论

本实验利用超高效液相色谱-三重四极杆质谱建立了30种胆汁酸的检测方法,并成功检测出大口黑鲈胆汁中19种胆汁酸以及草鱼胆汁中16种胆汁酸。该方法简单快速,灵敏度高,检测结果丰富了大口黑鲈和草鱼的胆汁酸谱库,为鱼类胆汁酸代谢的研究提供了数据支持。
